# Maternal weight status at conception predicts offspring body fat at age 11 years: population data from the Japan Kids Body Composition Study using dual-energy X-ray absorptiometry

**DOI:** 10.1186/s40101-024-00374-3

**Published:** 2024-10-19

**Authors:** Katsuyasu Kouda, Kumiko Ohara, Harunobu Nakamura, Yuki Fujita, Masayuki Iki

**Affiliations:** 1 Department of Hygiene and Public Health, Faculty of Medicine, Kansai Medical University, 2-5-1 Shin-Machi, Hirakata, Osaka 573-1010 Japan; 2 Department of Epidemiology for Community Health and Medicine, Kyoto Prefectural University of Medicine, 465 Kajii-Cho, Kawaramachi-Hirokoji, Kamigyo, Kyoto, 602-8566 Japan; 3 Department of Public Health, Faculty of Medicine, Kindai University, 377-2 Oono-Higashi, Osaka-Sayama, Osaka, 589-8511 Japan

**Keywords:** Adiposity, Children, Densitometry, Epidemiology

## Abstract

**Background:**

Maternal preconception overweight status has been reported to be associated with an increased risk of overweight offspring. However, there are no published population-based studies on the association between maternal preconception weight and offspring body fat measured by dual-energy X-ray absorptiometry (DXA). The present population-based retrospective cohort study aimed to examine the association between maternal weight at conception and offspring body fat measured by DXA.

**Methods:**

The source population consisted of 5th-grade students (1244 students aged 11 years) registered at 8 municipal elementary schools in Kitakata, Fukuroi, Hamamatsu, and Himeji in Japan. The present analyses included 964 participants who provided complete information. Maternal body mass index (BMI) at conception was calculated using records in the Mother and Child Health Handbook (MCHH). Offspring body fat at age 11 years was measured with the same QDR-4500A DXA instrument in a mobile test room that was brought to each school. With regard to the prediction of excess fat in offspring, the area under the curve (AUC) calculated with receiver operating characteristic curve analysis was used to quantify the diagnostic accuracy of maternal BMI at conception.

**Results:**

Adjusted odds ratios for excess body fat in offspring of the overweight mother group (odds ratios, 4.93 to 15.32) were significantly higher than those in the non-overweight mother group. For the prediction of excess offspring fat, AUCs and 95% confidence intervals for maternal BMI at conception were greater than 0.5.

**Conclusion:**

Maternal overweight status at conception might be a risk factor for excess body fat in offspring. Maternal BMI values calculated using MCHH data have the potential to distinguish between the presence and absence of excess fat in the next generation.

## Background

Obese children are at higher risk of being obese as adults, adult obesity is associated with an increased risk of morbidity, and overweight children are more likely to have diabetes and coronary heart disease in adulthood [1]. Recent systematic reviews and meta-analyses have identified a significant association between maternal preconception weight status and child weight, and have concluded that a higher maternal pre-pregnancy body mass index (BMI) is associated with an increased risk of higher offspring BMI [2, 3]. Poor nutrition and obesity are rife among women of reproductive age, and differences between high-income and low-income countries have become less distinct [4].

Although overweight status and obesity are defined as abnormal or excess fat accumulation that may impair health [5], most previous studies used BMI rather than body fat to define a child’s overweight status and obesity as an outcome variable [2, 3]. In children, weight gain is usually based on fat-free mass, rather than fat mass. Accordingly, BMI and other anthropometric surrogate measures are relatively poor predictors of metabolic risk. Therefore, body composition measurements that assess the relative proportion of adipose tissue, muscle, and bone are often considered more meaningful [6]. Moreover, the evaluation of body fat in mid- and late-childhood, rather than just the evaluation of body weight alone, is needed to predict the development of cardiometabolic disease in adulthood [6, 7].

To assess fat mass (FM) and fat-free mass (FFM), air displacement plethysmography, bioelectrical impedance analysis, hydrostatic weighing, and dual-energy X-ray absorptiometry (DXA) are commonly used [8]. Among these methods, DXA is a simple and safe technique that can be used for children and adults [9]. In addition, the precision of DXA measurements is excellent [9]. According to a recent systematic review, the validity of estimating body composition by DXA is superior compared with bioelectrical impedance analysis [10]. DXA is widely considered a valid method for measuring body FM, and DXA devices are commonly used in large-scale studies as well as in clinical and laboratory settings [8]. The use of DXA, and fat indices calculated based on it, may more clearly show the impact of maternal BMI at conception. Offspring body fat measured by DXA could be more informative for understanding the importance of maternal health and subsequent offspring outcomes.

There are no previous reports regarding the association between maternal preconception weight and childhood FM measured by DXA in a population-based cohort study. The present study aimed to examine the association between maternal weight at conception and DXA-detected FM in offspring at age 11 years in a large-scale population-based retrospective cohort study.

## Methods

### Study design and participants

The present study is a retrospective cohort study using population data from the Japan Kids Body Composition Study [11, 12]. The source population from which participants were recruited consisted of all 5th-grade students (age 11 years) registered at 8 municipal elementary schools in Japan (Shiokawa Elementary School, Dojima Elementary School, Ubado Elementary School, and Komagata Elementary School in Kitakata City in 2013; Fukuroi-kita Elementary School in Fukuroi City in 2009; Aritama Elementary School and Sekishi Elementary School in Hamamatsu City in 2008, 2009, 2010, and 2011; and Itohiki Elementary School in Himeji City in 2020). Of the source population of 1244 students, 964 who provided complete information were included in the present analysis (77.5% of the source population; 470 girls and 494 boys).

### Predictors and covariates

Maternal weight at conception was obtained from the Maternal and Child Health Handbook (MCHH), which serves as a record book to monitor maternal health and tracks the child’s health and growth [13]. The MCHH is distributed by local governments in Japan when pregnancies are registered, and coverage of the MCHH is almost 100% [14]. Records in the MCHH were conveyed to us by the parents or guardians of the children. BMI was calculated as body weight (kg) divided by height squared (m^2^). The BMI cut-off point for overweight status for the mother was 25 kg/m^2^.

### Outcome variables

All FM measurements at age 11 years were conducted with the same DXA instrument (QDR-4500A, Hologic Inc., Bedford, MA, USA) in a mobile test room that was brought to each elementary school. All FM measurements were also performed by the same experienced radiological technologist. Quality control of the DXA scanner was performed using phantoms supplied by the manufacturer, and the intra-machine variation calculated from 11 measurements with 2 volunteers was 3.0% (coefficient of variation) [12]. The between-center coefficient of variation was reported to be 5.6% [15]. Children removed all metal objects (e.g., zippers, belts, snaps, underwire bras) and their shoes, and laid flat on the scanner table during the DXA examination [12]. To assess adiposity, fat mass index (FMI) and body fat percentage (BFP) were used in the present study. FMI is a height-normalized index, is independent of overall body size, and is calculated as whole-body FM divided by height squared [16]. BFP corresponds to total FM divided by total body mass, multiplied by 100. Excess body fat was defined as FMI > 85th or 95th percentiles and as BFP > 85th or 95th percentiles [17, 18].

Anthropometric measurements at age 11 years were performed in light clothing and without shoes and hats. Participants were asked to undo hairstyles and remove hair accessories that may interfere with height measurement. Body height was measured in an upright position with both heels close together; the back, buttocks, and heels touching the scale post of the stadiometer; both arms hanging down; and the head in the eyes-ears horizontal position [19]. Height and waist circumferences were measured to the nearest 0.1 cm. Body weight was measured to the nearest 0.1 kg. Overweight and underweight children were identified using sex- and age-specific international cut-offs for BMI (overweight 20.66 kg/m^2^ for girls and 20.51 kg/m^2^ for boys; underweight 15.03 kg/m^2^ for girls and 14.96 kg/m^2^ for boys) [20].

### Statistical analysis

Data were analyzed using SPSS Statistics Desktop for Japan, Version 26 (IBM Japan, Ltd., Tokyo, Japan). Unpaired *t*-test or Fisher’s exact test was used to assess differences between non-overweight and overweight mothers at conception. *P* < 0.05 was considered statistically significant. Logistic regression analysis was performed to examine the association of maternal weight at conception with DXA-detected FM in offspring at age 11 years. Logistic regression analysis incorporated potential confounders including variables that could be theorized, such as gestational age at birth [21] and maternal age [22], in accordance with a previous report [23]. Duplicate variables, such as those for maternal height, weight, and BMI, were excluded from the same multivariate analysis. The 95% confidence interval (CI) of the odds ratio (OR) not containing the value 1 was considered statistically significant. The predictive performance of maternal BMI at conception, specifically with regard to its capacity to discriminate between the presence and absence of excess offspring FM at age 11 years, was evaluated using receiver operating characteristic (ROC) curve analysis [24]. The area under the curve (AUC) was used to quantify the diagnostic accuracy of maternal BMI at conception [24]. The 95% CI of AUC not containing the value 0.5 was considered statistically significant. Generally, an AUC value of 0.5 indicates that the ROC curve will fall on the diagonal, and values of 0.7 to 0.8 are considered acceptable [25].

## Results

Minimum, maximum, mean, and standard deviation values of maternal BMI at conception were 14.48 kg/m^2^, 35.56 kg/m^2^, 20.08 kg/m^2^, and 2.45 kg/m^2^, respectively. Table 1 shows participant characteristics classified by maternal weight at conception. For both girls and boys at age 11 years, the offspring of overweight mothers had a larger BMI, waist-to-height ratio (WtHR), FMI, and BFP compared with those of non-overweight mothers. Table 2 shows crude and adjusted ORs for excess fat status in offspring. All ORs for FMI at age 11 years > 85th or 95th percentiles in the offspring of overweight mothers were significantly higher compared with those in the offspring of non-overweight mothers for both sexes. Similarly, all ORs for BFP at age 11 years > 85th or 95th percentiles in the offspring of overweight mothers were significantly higher compared with those in the offspring of non-overweight mothers for both sexes.

**Table 1:** Participant characteristics

	Maternal weight status at conception		
	Non-overweight, *N* = 920	Overweight, *N* = 44	*P*-value^a^
Girls, *N* = 470			
Maternal characteristics			
Age at birth, years	29.0 (4.4)	29.5 (4.2)	0.671
Height at preconception, cm	157.5 (5.0)	159.4 (5.7)	0.141
Weight at preconception, kg	48.8 (5.6)	69.6 (7.2)	< 0.001
BMI at preconception, kg/m^2^	19.7 (1.9)	27.3 (1.8)	< 0.001
Gestational age at birth, weeks	38.7 (1.8)	39.1 (0.9)	0.432
Offspring characteristics at age 11 years			
Age, years	11.2 (0.3)	11.1 (0.3)	0.584
Height, cm	143.7 (6.7)	146.9 (6.0)	0.058
Weight, kg	35.2 (6.6)	44.4 (13.1)	0.014
BMI, kg/m^2^	16.9 (2.2)	20.3 (4.7)	0.012
BMI >overweight cut-off^b^, *N* (%)^c^	29 (6)	6 (38)	< 0.001
BMI <underweight cut-off^b^, *N* (%)^c^	69 (15)	2 (13)	1.000
Waist circumference, cm	62.6 (6.1)	71.6 (11.8)	0.008
WtHR	0.44 (0.04)	0.49 (0.07)	0.010
WtHR > 0.5, *N* (%)^c^	29 (6)	5 (31)	0.004
FM, kg	7.6 (3.0)	12.3 (7.1)	0.019
FMI, kg/m^2^	3.6 (1.3)	5.6 (2.9)	0.019
BFP, %	20.5 (4.8)	25.7 (7.7)	0.017
Boys, *N* = 494			
Maternal characteristics			
Age at birth, years	29.0 (4.4)	28.0 (4.1)	0.229
Height at preconception, cm	157.5 (5.3)	156.3 (5.3)	0.232
Weight at preconception, kg	49.2 (5.6)	66.1 (7.1)	< 0.001
BMI at preconception, kg/m^2^	19.8 (1.9)	27.1 (2.6)	< 0.001
Gestational age at birth, weeks	38.8 (1.5)	38.4 (2.9)	0.419
Child characteristics at age 11 years			
Age, years	11.2 (0.3)	11.0 (0.3)	0.012
Height, cm	141.7 (6.5)	142.0 (5.6)	0.832
Weight, kg	34.8 (7.2)	41.9 (11.0)	0.002
BMI, kg/m^2^	17.2 (2.5)	20.6 (4.6)	< 0.001
BMI >overweight cut-off^b^, *N* (%)^c^	53 (11)	14 (50)	< 0.001
BMI <underweight cut-off^b^, *N* (%)^c^	57 (12)	1 (4)	0.232
Waist circumference, cm	63.4 (7.4)	72.5 (12.8)	< 0.001
WtHR	0.45 (0.04)	0.51 (0.08)	< 0.001
WtHR > 0.5, *N* (%)^c^	53 (11)	14 (50)	< 0.001
FM, kg	7.0 (3.6)	12.7 (7.2)	< 0.001
FMI, kg/m^2^	3.4 (1.6)	6.2 (3.3)	< 0.001
BFP, %	18.7 (5.9)	27.8 (8.9)	< 0.001

*N* number, *BMI* body mass index, *WtHR* waist to height ratio, *FM* fat mass, *FMI* fat mass index, *BFP* body fat percentage

^a^ *P*-value calculated from the unpaired *t*-test or Fisher’s exact test

^b^Determined using age- and sex-specific BMI cut-off points

Values represent mean (standard deviation), except for ^c^ *N* of children (% of children)

**Table 2:** Odds ratios and 95% confidential intervals for excess fat status in offspring

	Girls, *N*=470		Boys, *N*=494	
Maternal weight status at conception	OR	95% CI	OR	95% CI
FMI at age 11 years >95th percentile in offspring				
Unadjusted analysis				
Non-overweight mother	1.00		1.00	
Overweight mother	5.01	(1.32-18.99)	14.24	(5.53-36.65)
Adjusted analysis^a^				
Non-overweight mother	1.00		1.00	
Overweight mother	4.93	(1.29-18.77)	15.32	(5.82-40.30)
FMI at age 11 years >85th percentile in offspring				
Unadjusted analysis				
Non-overweight mother	1.00		1.00	
Overweight mother	6.32	(2.29-17.46)	9.38	(4.23-20.82)
Adjusted analysis^a^				
Non-overweight mother	1.00		1.00	
Overweight mother	6.52	(2.35-18.08)	9.86	(4.39-22.14)
BFP at age 11 years >95th percentile in offspring				
Unadjusted analysis				
Non-overweight mother	1.00		1.00	
Overweight mother	5.01	(1.32-18.99)	11.25	(4.31-29.37)
Adjusted analysis^a^				
Non-overweight mother	1.00		1.00	
Overweight mother	5.23	(1.37-20.03)	12.97	(4.79-35.10)
BFP at age 11 years >85th percentile in offspring				
Unadjusted analysis				
Non-overweight mother	1.00		1.00	
Overweight mother	6.32	(2.29-17.46	13.18	(5.79-29.98)
Adjusted analysis^a^				
Non-overweight mother	1.00		1.00	
Overweight mother	6.17	(2.23-17.10	13.83	(6.01-31.87)

Logistic regression analysis was performed to examine the association of maternal weight with offspring fat

*N* number, *OR* odds ratio, *CI* confidence interval, *FMI* fat mass index, *BFP* body fat percentage

^a^Adjusted for maternal age at delivery and gestational age at birth

**Fig. 1:**
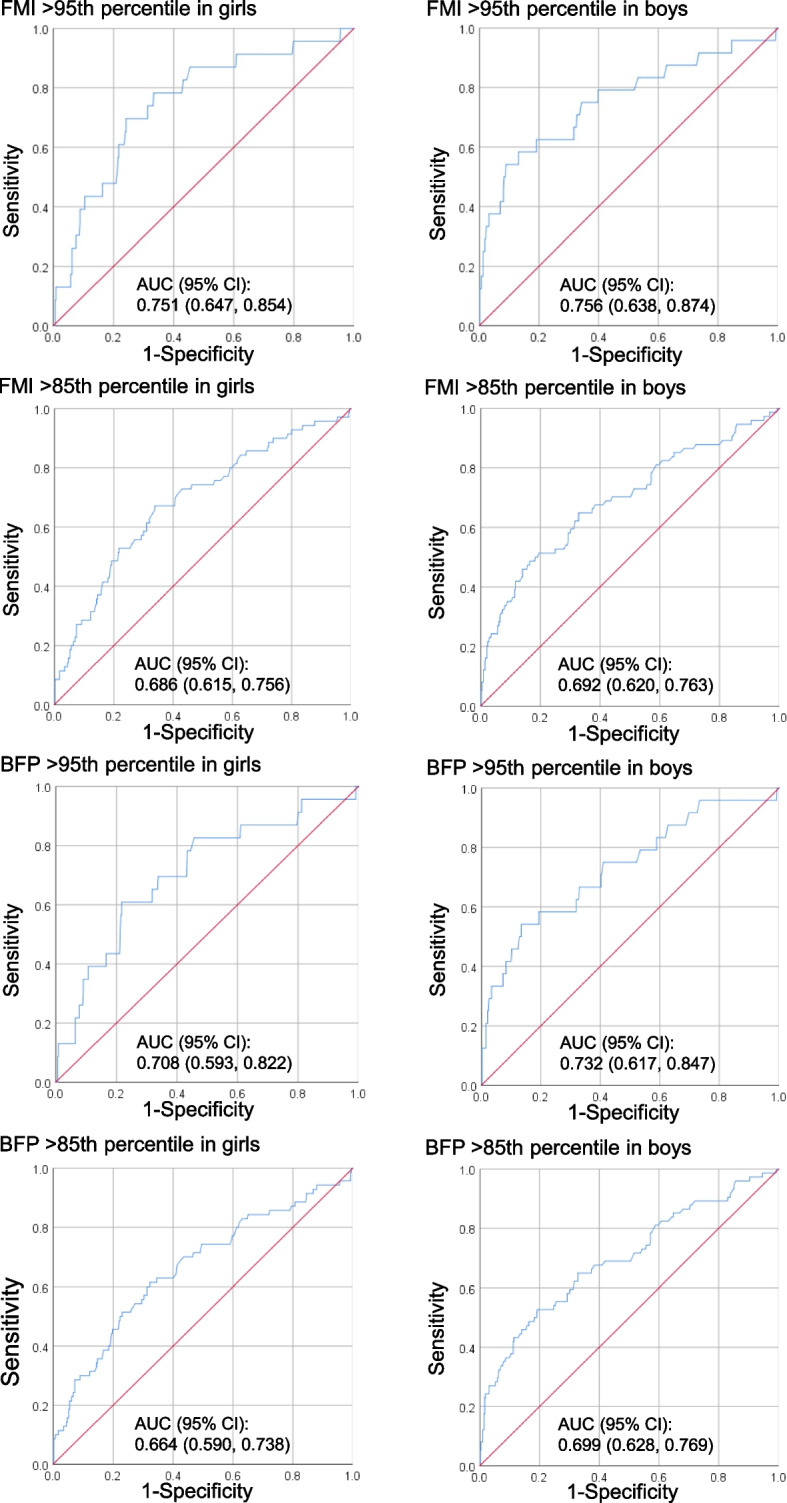
Diagnostic performance of maternal body mass index at conception to predict offspring excess fat. Excess body fat was defined as fat mass index (FMI) > 85th or 95th percentiles and as body fat percentage (BFP) > 85th or 95th percentiles. Receiver operating characteristic curve analysis was performed. The area under the curve (AUC) with a 95% confidence interval (CI) was used to quantify the diagnostic accuracy of maternal body mass index at conception

Figure 1 shows ROC curves for maternal BMI at conception to distinguish between offspring with and without excess fat. All AUCs for FMI at age 11 years > 85th or 95th percentiles in offspring were significantly larger than 0.5 for both sexes. Similarly, all AUCs for BFP at age 11 years > 85th or 95th percentiles in offspring were significantly larger than 0.5 for both sexes.

## Discussion

To our knowledge, the present study is the first to assess the association between maternal weight at conception and DXA-measured body fat in offspring. The present population-based retrospective cohort study found that maternal weight at conception was positively associated with offspring body fat at age 11 years, and that maternal overweight status at conception might be a risk factor for excess body fat in offspring. Furthermore, ROC curve analysis showed that AUCs and 95% CIs for BMI at conception and excess fat at age 11 years were greater than 0.5, and that maternal BMI values calculated using MCHH data have the potential to distinguish between the presence and absence of excess fat in offspring at age 11 years. In Japan, the MCHH is distributed by local governments, coverage of the handbook is almost 100%, and most parents keep the handbook until their children are married [14]. At present, the MCHH program is being introduced in more than 20 countries, including both developing and developed countries [14]. Therefore, maternal BMI at conception calculated from height and weight records in the MCHH could serve as useful information that may contribute to the prevention of excess fat in offspring in mid- and late-childhood.

Recent systematic reviews and meta-analyses have identified a significant association between maternal preconception weight status and child weight [2, 3], and reported that, as compared to mothers with normal weight (18.5–24.9 kg/m^2^), maternal overweight status (25.0–29.9 kg/m^2^) and obesity (≥ 30.0 kg/m^2^) were associated with higher risks of overweight/obese offspring (ORs for overweight/obesity defined using WHO cutoffs in late childhood: 2.28 and 4.47 for maternal overweight status and obesity, respectively) [3]. Thus, ORs for excess body fat in the offspring of overweight mothers in the present study appeared to be larger compared with ORs for excess BMI in the offspring of overweight mothers in the previous meta-analysis. These results suggest that the use of DXA, and fat indices calculated based on it, may more clearly show the impact of a mother’s BMI at conception.

The mechanism underlying the association between maternal weight at conception and offspring body fat was not explored in the present study. However, causal effects of maternal obesity on offspring obesity, which are mediated at least partly through changes in epigenetic processes including alternations in DNA methylation in utero, have been suggested in experimental studies [26]. Initial research linking developmental influences with cardiometabolic disorders in later life focused on the effects of fetal undernutrition, while accumulating evidence indicates that exposure to maternal obesity also leads to an increased risk of disease in offspring [26]. On the other hand, the family is an important social context where children learn and adopt eating behaviors, and parents play the role of health promoters and educators in the lives of children [27]. The preconception period can be a sensitive phase in life, when health behaviors affecting diet, exercise, and obesity, along with smoking and drinking, become established [4]. A previous study reported that maternal body weight is more strongly associated with pubertal weight in offspring than birth weight or infantile weight and that the relationship between maternal pre-pregnancy weight and offspring weight strengthens as children develop [28]. These results suggest that the association between maternal weight and offspring weight in adolescence might be explained by the extrauterine nutritional environment, which is attributed to maternal lifestyle [28].

If mothers are exposed to an obesogenic environment that promotes an unhealthy lifestyle, then their children are likely to be exposed to the same obesogenic factors [2]. Indeed, a recent systematic review and meta-analysis concluded that a number of parental behaviors are strong correlates of child food consumption behavior [27]. Another systematic literature review found that there was fairly consistent evidence for the association of maternal stress with children’s lower physical activity and higher sedentary behavior [29]. Evidence for the association between parental behavior and children’s physical activity or sedentary behavior was also discussed in another systematic review [30]. These results indicate that the adverse consequences of poor nutrition combined with obesity, rife in women of reproductive age, may extend across generations [4].

Previous systematic reviews have reported that overweight status and obesity in childhood and adolescence have adverse consequences on premature mortality and physical morbidity in adulthood [31], and that prevention of childhood obesity should remain a priority for public health interventions to prevent negative health outcomes during childhood as well as reducing the burden of adult obesity [32]. The US Preventive Services Task Force recommends that clinicians screen for obesity in children and adolescents 6 years and older and offer or refer them to comprehensive, intensive behavioral interventions to promote improvements in weight status [33]. Based at least in part on this, we obtained information on excess body fat in 11-year-old children as an outcome variable in the present study.

A strength of the present study is that large-scale population-based participants were recruited from residents of a defined location. This contrasts with a recently published study which had a relatively small sample population from hospital-based recruitment [23]. A population-based sample, in contrast to a sample based on hospital recruitment [23], is the ideal setting for carrying out unbiased evaluations of relationships, not only of confounders to exposures and outcomes but also among any other variables of interest [34]. In the present study, we used a DXA instrument in a mobile test room that was brought to each elementary school in all areas of the study and obtained population-based data for body fat. In contrast, although a DXA instrument is generally available in a hospital, it is difficult to obtain population-based data in that setting. Indeed, the hospital-based study mentioned above reported a lack of DXA data for 38% of eligible offspring [23]. In addition, the sample size of the present study was sufficient for multivariate logistic regression and ROC curve analyses. The single-center study design also has advantages over a multi-center design, because there is no need for inter-center calibration of DXA measurements. Finally, the advantages of retrospective cohort studies are that exposure to risk factors is recorded before the occurrence of the outcome, and they allow for the temporal sequence of risk factors and outcomes to be assessed [35].

The present study also has potential limitations. First, study areas were not randomly selected from throughout Japan. Specifically, the source population consisted of all 5th-grade students registered at four municipal schools in Fukushima (the southernmost prefecture of the Tōhoku Region), three municipal schools in Shizuoka (located halfway between Tokyo and Osaka), and one municipal school in Hyogo (located in the Kansai Region). Since these students are not fully representative of the general population of Japanese children, caution is required when generalizing the results. However, since there are no private schools in these study areas, almost all children living in these areas are enrolled in the municipal schools targeted in this study. In addition, mean height/weight measurements of girls and boys at age 11.2 years in Japanese national surveys were 144.0 cm/37.3 kg and 142.5 cm/36.6 kg, respectively [36], and those in the present study were 143.8 cm/35.5 kg and 141.7 cm/35.2 kg, respectively, showing negligible differences in anthropometric variables between the present study population and the Japanese national population. Second, fat accumulation is strongly related to sexual maturity, especially in pubertal children. However, the sexual development status of participants was not taken into account in the present study. Third, we did not have information on various parameters that could influence body composition, including the number of births experienced, paternal BMI, family income, and parental education.

Maternal weight at conception was positively associated with offspring body fat at age 11 years in Japan. Overweight status at conception might be a risk factor for excess body fat in offspring. In addition, ROC curve analysis showed that maternal BMI values calculated using MCHH data have the potential to distinguish between the presence and absence of excess fat in offspring in mid- and late-childhood. Maternal weight records thus could serve as useful information that may contribute to the prevention of excess fat in the next generation.

## Data Availability

The data that support the findings of the current paper are available from the corresponding author [KK] upon reasonable request.

## Abbreviations

AUC: Area under the curve
BFP: Body fat percentage
BMI: Body mass index
CI: Confidence interval
DXA: Dual-energy X-ray absorptiometry
FM: Fat mass
FMI: Fat mass index
FFM: Fat-free mass
MCHH: Mother and Child Health Handbook
N: Number
OR: Odds ratio
ROC: Receiver operating characteristic
WtHR: Waist to height ratio
